# Suppression of host nocifensive behavior by parasitoid wasp venom

**DOI:** 10.3389/fphys.2022.907041

**Published:** 2022-08-12

**Authors:** Amit Rana, Stav Emanuel, Michael E. Adams, Frederic Libersat

**Affiliations:** ^1^ Department of Life Sciences and Zlotowski Center for Neurosciences, Ben Gurion University of the Negev, Be’er Sheva, Israel; ^2^ Department of Molecular, Cell, and Systems Biology, University of California, Riverside, Riverside, CA, United States; ^3^ Department of Entomology, University of California, Riverside, Riverside, CA, United States

**Keywords:** nociceceptive modulation, parasitoid wasp, central complex, interneurons, venom, noxious stimulus, cockroach

## Abstract

The parasitoid wasp *Ampulex compressa* envenomates the brain of its host the American cockroach (*Periplaneta americana*), thereby making it a behaviorally compliant food supply for its offspring. The target of venom injection is a locomotory command center in the brain called the central complex. In this study, we investigate why stung cockroaches do not respond to injuries incurred during the manipulation process by the wasp. In particular, we examine how envenomation compromises nociceptive signaling pathways in the host. Noxious stimuli applied to the cuticle of stung cockroaches fail to evoke escape responses, even though nociceptive interneurons projecting to the brain respond normally. Hence, while nociceptive signals are carried forward to the brain, they fail to trigger robust nocifensive behavior. Electrophysiological recordings from the central complex of stung animals demonstrate decreases in peak firing rate, total firing, and duration of noxious-evoked activity. The single parameter best correlated with altered noxious-evoked behavioral responses of stung cockroaches is reduced duration of the evoked response in the central complex. Our findings demonstrate how the reproductive strategy of a parasitoid wasp is served by venom-mediated elimination of aversive, nocifensive behavior in its host.

## 1 Introduction

The jewel wasp (*Ampulex compressa*) uses mechano-sensors on its stinger to locate and inject venom directly into the head ganglia of its cockroach host (*Periplaneta americana*) (Gal et al., 2014). The wasp envenomates both cerebral and gnathal ganglia (CRG and GNG, respectively) located in the cockroach head capsule (Haspel et al., 2003). To provide a fresh food supply for its offspring, the wasp venom induces a behavioral sequence in the host beginning with an intense bout of grooming (lasting ∼20–30 min), followed by onset of a long-lasting (5–7 days) sedentary condition termed “hypokinesia.” In this respect, the jewel wasp sting is unusual compared to the vast majority of parasitoid wasps, that completely paralyze their host (Gal and Libersat, 2008). In addition, and surprisingly so, stung cockroaches do not respond to several harmful threats during the manipulation process. Specifically, the wasp breaks off both antennae to drink hemolymph from the stumps. The wasp also pulls strongly on one of the antennae while dragging the stung cockroach to the nest. Finally, *A. compressa* larva hatches approximately 3 days after oviposition and pierces the soft cuticle of the cockroach near the base of the leg to feed on hemolymph with no reaction from the cockroach. Given these observations, during the manipulation process, the cockroach experiences strong noxious stimuli, which jeopardize integument (cuticle) integrity. Yet, it does not try to avoid the noxious stimuli or engage in nocifensive behavior (escape from a noxious stimulus). How does action of the wasp venom interfere with the normal responses to such noxious stimuli? Based on these observations, we hypothesize that envenomation of the cerebral ganglia impedes CNS processing of noxious stimuli.

In the 1980s and early 1990s, many studies explored the existence and possible role of an opioid system in invertebrates and, particularly relevant here, in insects (Huber et al., 1990). Opioid-like substances are known to have “analgesia”-like effects that modulate the threshold for escape in insects (Huber et al., 1990). Furthermore, opioid-like neurons and fibers have been identified in cockroaches (Verhaert and De Loof, 1985). Of direct relevance here, Gavra and Libersat (2010) showed that cockroaches injected systemically with different opioid receptor agonists or antagonists showed an increase or decrease, respectively, of the escape response threshold to noxious stimuli. In these experiments, they examined the escape behavior of stung and un-stung cockroaches in response to noxious electric foot shock (Gavra and Libersat, 2010). Furthermore, an antennal heart preparation, which bears enkephalin-like receptors (the equivalent assay to that of the pig ileum), was used to evaluate the presence of opiate-receptor binding molecules in the wasp venom. In un-stung cockroaches, external application of crude venom completely inhibited antennal heart contractions. This inhibitory effect was readily reversed by naloxone, a non-specific opioid high-affinity receptor antagonist (Gavra and Libersat, 2010). Additionally, Kaiser et al. (2019) identified one of the venom components as Neurotrimin/Opioid-binding protein/cell adhesion molecule (OBCAM).

Using radiolabeled venom in wasps, it was shown that the primary target of wasp venom is the central complex (CX) in the cerebral ganglia (Haspel et al., 2003). The CX is a multilayered structure with sensory processing and pre-motor functions and has been extensively studied in several insect species. As a sensory unit, central complex processes information related to polarized light (Heinze and Homberg, 2007; Heinze et al., 2009), deflection of the antenna (Ritzmann et al., 2008) and head direction estimation (Varga and Ritzmann, 2016). As a pre-motor center, the CX has a role in initiation and maintenance of locomotion (Guo and Ritzmann 2013; Martin et al., 2015). Furthermore, the firing rate of CX neurons in cockroaches is directly correlated with stepping rate (Bender et al., 2010) and walking can be modulated by electric stimulation of the CX. In addition, focal injection of procaine (a reversible Na + channel blocker) into the CX leads to decreased spontaneous walking (Kaiser and Libersat, 2015). More recently, the fan-shaped body, a subregion of the CX, has been implicated in regulation of nocifensive behavior in *Drosophila* (Hu et al., 2018). Specifically, activation of fan-shaped body neurons leads to the onset of nocifensive behavior. Taken together, these facts suggest a central role of the CX in processing nociceptive information. In the present work, we investigated nocifensive behavior of cockroaches stung by the jewel wasp and the location of nociceptive pathway impairment in the central nervous system. Furthermore, we recorded CX responses to the nociceptive stimuli *in vivo* before and after venom injection into the CX.

## 2 Materials and methods

### 2.1 Animals

Adult male cockroaches (*P. americana*) were raised in crowded conditions in plastic containers (50 × 50 × 70 cm) under a 12 h D: L cycle at 26°C. Water and food (cat chow) were provided *ad libitum*. Female wasps used for experiments were maintained at 25–30°C and humidity above 50%. Wasps were provided water and honey *ad libitum*.

### 2.2 Noxious stimuli

In the first series of experiments, we applied crude noxious stimuli that were as similar as possible to those inflicted by the wasp. Tactile stimuli to the abdomen and noxious stimuli (abdominal cuticle cuts or antennal acute ablations) were applied using scissors (for both tactile and noxious manual stimuli) to evaluate escape responses of un-stung vs. stung individuals. For electrophysiological analysis of nociceptive interneurons, we used the same custom-built device as described in Emanuel and Libersat (2019). The device is based on a step motor that drives a metal tip with changing temperatures mounted on a micromanipulator. This device was used to apply calibrated tactile and noxious stimuli of variable duration. Using this device, tactile or combined tactile and noxious heat stimuli (100°C; 3 s) were applied (referred to as “brief” stimuli). In addition, to distinguish between the tactile and noxious components of the stimulus, a “cold” probe was placed on the cuticle and, without removing the probe, it was heated to 100°C (referred to as “continuous” stimuli). Moreover, to apply pure noxious stimuli to the cockroaches, we used a focusable 450 nm, 500 mW blue dot laser diode module with a 12V TTL driver (JOLOOYO, China). The laser was positioned 15 cm above the cockroach’s abdomen and the laser beam was focused on a dot roughly 100 μm in diameter. Duration of the stimulus was controlled using an isolated pulse stimulator (A-M Systems, 2,100 model). Behavioral responses to noxious stimuli were measured in tethered cockroaches standing on a slippery glass platform covered with mineral oil. A photo-resistor was placed under the metathoracic leg while a light source was placed above the same leg to monitor leg movement (as described in Fouad et al., 1994; Emanuel and Libersat, 2019). Each recorded spike from the photo-resistor corresponds to a single leg step. Noxious stimuli were applied using either a heat probe or a laser beam (50–400 ms with 50 ms increments) on the cuticle of an abdominal segment. The laser stimulus was moved to a different location after every trial to avoid desensitization due to cuticle heating. This set-up was used to compare the escape response of stung cockroaches as compared to un-stung (control) cockroaches. Furthermore, involvement of cockroach head ganglia in nocifensive responses was tested by severing neck connectives surgically under cold anesthesia (referred to as “Neck Connective Cut”) and monitoring changes in escape responses to laser stimuli.

### 2.3 Venom injection

To test venom actions on the CX neuronal population, whole venom obtained as described previously (Kaiser and Libersat, 2015) was drawn into a glass pipette connected to a nanovolumetric injector (Drummond’s Nanoinject II). Throughout the process, the nano-injector needle was kept as close to ice as possible.

### 2.4 Electrophysiology

To establish if nociceptive information is carried along the stung cockroach central nervous system, extracellular recordings of projection interneurons were made as described previously in Emanuel and Libersat (2019). Electrical activity was recorded with an A-M Systems Model 1700 Differential AC Amplifier and sampled at 20 kHz *via* a CED Micro 1,401 analog-to-digital board (Cambridge Electronic Design). In addition, EMG responses of the metathoracic coxal depressor muscle were recorded as described in Emanuel and Libersat (2019). Since the coxal depressor muscle is controlled by only two excitatory motor neurons (Usherwood, 1962; Pearson and Iles, 1971), it was usually possible to discriminate between large amplitude EMG spikes indicating fast motor neuron (Df) activity and smaller amplitude EMG spikes indicative of slow motor neuron (Ds) activity. For chronic CX recordings, neuronal activity was recorded using a TDT Rz5 bio amp processor amplifier (Tucker Davis Technologies, United States) suitable for multi-channel recording.

Involvement of the CX in the nociceptive pathway was tested by making chronic CX recordings from cockroaches fixed on a horizontal plate with insect pins and a U-shaped neck pin placed between head and thorax to reduce hemolymph pulsations. Most head cuticle was removed along with the antennal lobe, ocelli and most of the jaw. Once the cerebral ganglia were exposed, fat tissue and tracheae were removed using fine forceps. Cerebral ganglia were stabilized for recording with a metal wire platform placed beneath, after which the neural sheath was gently peeled off using fine forceps (Saha et al., 2013). A custom built two-channel monopolar electrode (Formvar coated 37 µm NiCr from A-M Systems) was inserted into the CX using a micromanipulator (Guo et al., 2014) and a ground electrode (75 µm silver wire from A-M systems) was inserted into the hemolymph through a hole in the pronotum. Recordings were initiated upon detection of spontaneous spikes (Guo et al., 2014). About 20 min after initiation of recording, a laser was used to apply 2 sets of stimuli, an ineffective 100 ms stimulus set (no nociceptive response reported to this stimulus in the behavioral experiments) and an effective 400 ms nociceptive stimulus set (strong nociceptive response reported in the behavioral experiments). A nanovolumetric injector (Drummond Nanoinject II) delivered a total of 27 nl of crude venom into the CX in 3 installments, each comprising 9 nl. An hour after venom injection, neuronal responses to both sets of nociceptive laser stimuli were assayed again. At the end of each experiment, a lesion was induced at the site of the recording electrodes by applying a 10 µA current for 5 s to confirm placement.

### 2.5 Analysis and statistics

In the behavioral assay to noxious stimuli *via* a heat probe, “escape duration” was measured in seconds from the raw data. For testing escape behavior in response to noxious stimuli *via* laser, the total number of steps was also measured along with escape duration in un-stung, stung and neck-connectives cut cockroaches. To measure strength of response in extracellular recordings, a root mean square (RMS) procedure was applied to the waveform data using Spike2 version 5.05 software (Cambridge Electronic Design). RMS was calculated by summing the square of each data point, dividing the sum by the number of data points and then taking the square root of the result. Then the area beneath the resulting waveform data was measured (“RMS Area”). To normalize response strength, RMS area measurements before the stimulus was subtracted from the RMS area measurements after the stimulus. This technique of analysis is often used for quantifying EMGs and less often to quantify extracellular recordings. But regarding the later, RMS measurement was preferred over spike counting because it is a better measure of neuronal response strength that often includes compound spikes. Hence, we applied RMS analysis to compare power of the responses of the sensory nerve, the interneurons, and the muscles. Electrophysiological recordings from the CX were made to monitor changes in the activity of a small number of neurons, which allows distinguishing between the activity of different neurons (or “firing units”) in one recording. Thus, the CX recordings were analyzed much more extensively by using a custom-built spike2 script. The spikes were detected using a manually defined threshold. The potential spikes were then sorted based on their unique wave shape. Spikes having 70% or greater similarity were defined as the same unit. Furthermore, principal component analysis assisted clustering was performed on these identified units to obtain the final units. Timing of each isolated spike was used to plot the peri-stimulus time histogram (PSTH) to the laser stimulus. The PSTH starts 500 ms (spontaneous activity) before stimulus onset and lasts until 1500 ms (evoked activity) after stimulus onset. These 2-s recordings were then divided into 100 ms as well as 250 ms time bins for each cockroach and each stimulus type. Duration of the response to the nociceptive stimulus was calculated as the period of significantly elevated evoked activity compared to the baseline (spontaneous activity). The total evoked activity was determined by summation of neuronal spikes within one second of the response window. Stimulus-response latency was calculated as the time between onset of the stimulus and peak neuronal response (maximum number of spikes within a 100 ms time bin). All statistical tests were performed either using SigmaPlot 13.0 or GraphPad Prism 7.0 (GraphPad Software, Inc., San Diego, CA) software. If data were not normally distributed according to the Shapiro–Wilk test, non-parametric statistical tests were used. Statistical significance was determined using Student’s t-test, paired t-tests, Mann–Whitney rank-sum tests, or Wilcoxon signed-rank tests. One-way ANOVA was used to compare averages among the three groups. For all pairwise multiple comparison procedures, the Holm-Sidak method was used. RM ANOVA on ranks was performed to determine the statistical significance in the firing rate due to laser stimulus. Dunnett’s test was performed to compare the baseline-firing rate with laser-evoked firing rate at various time points up to 1,500 ms after stimulus onset. All electrophysiological data points are presented as mean ± SEM.

## 3 Results

### 3.1 Nocifensive behavior is suppressed in stung cockroaches

**FIGURE 1:**
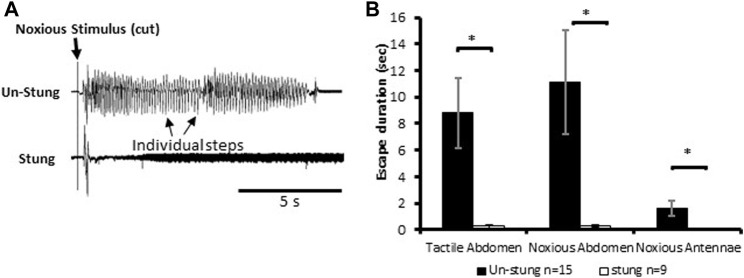
Noxious stimulus does not induce a typical nocifensive escape response in stung individuals. **(A)** Leg movements (individual steps, which represent escape) of un-stung (upper trace) and stung (lower trace) cockroaches in response to a manual noxious abdominal stimulus. Stimulus onset is indicated with a vertical line. **(B)** The escape duration of stung cockroaches is significantly lower than that of un-stung cockroaches in response to all types of stimuli. Bars represent means ± SEM, significance is indicated with an asterisk.

Stimuli consisting of a tactile stimulus to an abdominal segment or physical damage to antenna or an abdominal segment elicit escape responses in tethered un-stung cockroaches (Figure 1). All types of applied stimuli evoke a fast-running escape response in un-stung cockroaches (n = 15; mean escape duration in sec ±SEM: tactile abdomen 8.82 ± 2.63, noxious abdomen 11.11 ± 3.91 and noxious antennae 1.64 ± 0.59; Figure 1B). By comparison, stung cockroaches show a significant decrease in running duration as compared to un-stung cockroaches (Figure 1B; tactile abdomen *p* = 0.003, noxious abdomen *p* = 0.012, noxious antennae *p* = 0.031, Mann-Whitney rank-sum test). Un-stung cockroaches (n = 15) respond in robust fashion to 3 different types of stimuli (tactile abdomen, noxious abdomen, and noxious antennae), while stung cockroaches (n = 9) respond weakly, if at all, with a brief startle to tactile and noxious stimuli (means in sec ±SEM: tactile abdomen 0.28 ± 0.15, noxious abdomen 0.29 ± 0.09, noxious antennae 0.00 ± 0.00; Figure 1B). No significant difference was found when comparing escape duration of stung cockroaches to different types of stimuli (tactile abdomen or noxious abdomen). These data demonstrate an almost complete suppression of nocifensive behavior in stung cockroaches.

### 3.2 Nociceptive input to the brain is intact in stung cockroaches

**FIGURE 2:**
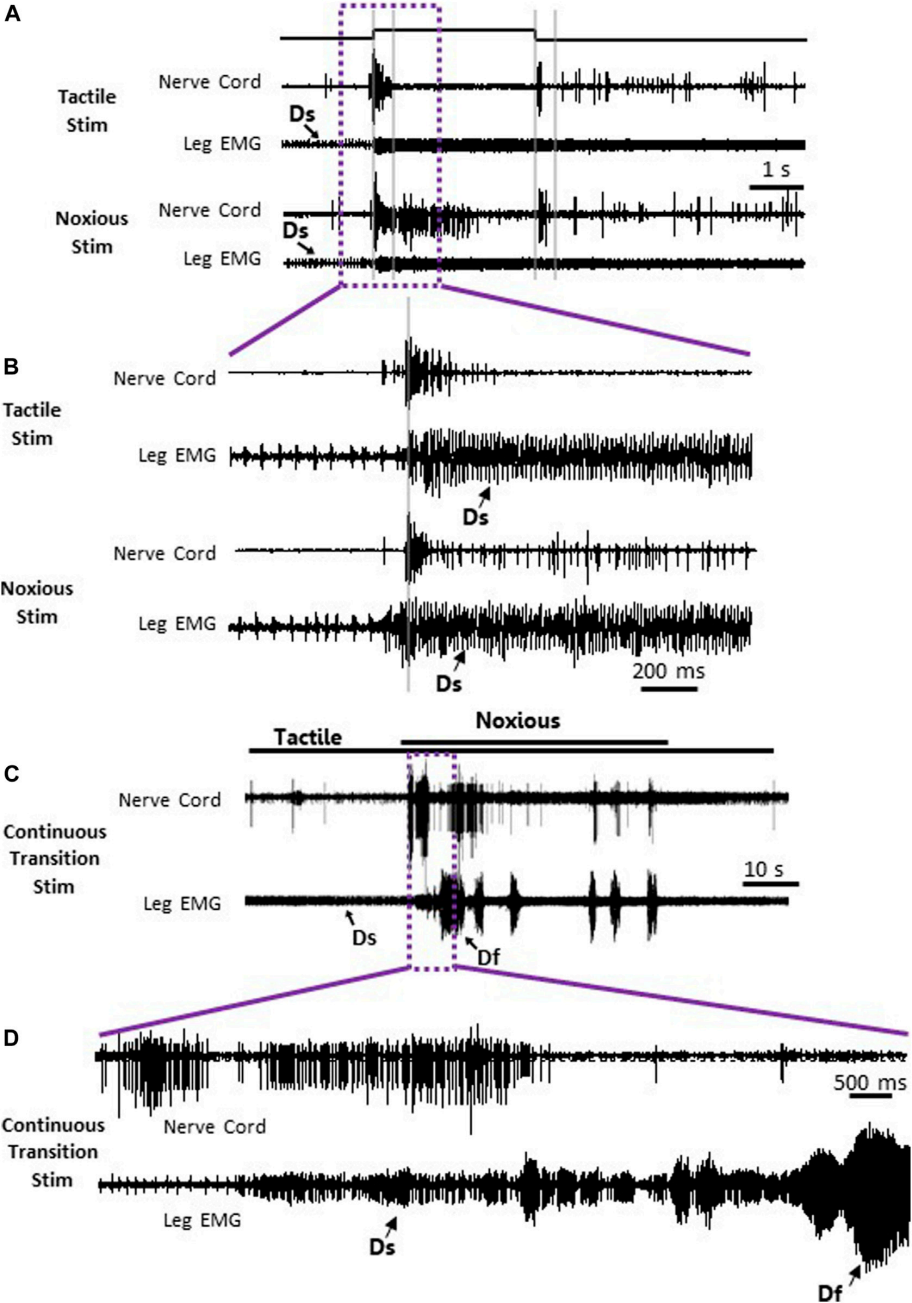
Nociceptive and tactile information is carried forward by post-synaptic interneurons in the nerve cord of stung cockroaches. **(A)** Representative example recordings of post-synaptic interneurons in the nerve cord (upper trace of each stimulus example) and the leg EMG (lower trace of each stimulus type) following a brief (3 s) tactile (two upper traces) and noxious (two lower traces) stimuli. Vertical grey lines represent a time division (0–0.4, 0.4–3, and 3–3.4 s) corresponding to onset, duration, and offset of the stimulus, respectively. Activity of the slow motor neurons (Ds) is indicated on the leg EMG traces. **(B)** Enlarged view of traces in purple box in panel **(A)**. **(C)** Representative example of simultaneous recordings from post-synaptic interneurons in the nerve cord (upper trace) and the leg EMG (lower trace) following a continuous transition between tactile and noxious stimuli. Stimulus duration is indicated with horizontal lines (upper line: noxious; lower line: tactile). **(D)** Enlarged view of traces in purple box in panel **(C)**. Activity of the fast and slow motor neurons (Df and Ds, respectively) is indicated on the leg EMG traces.

**FIGURE 3:**
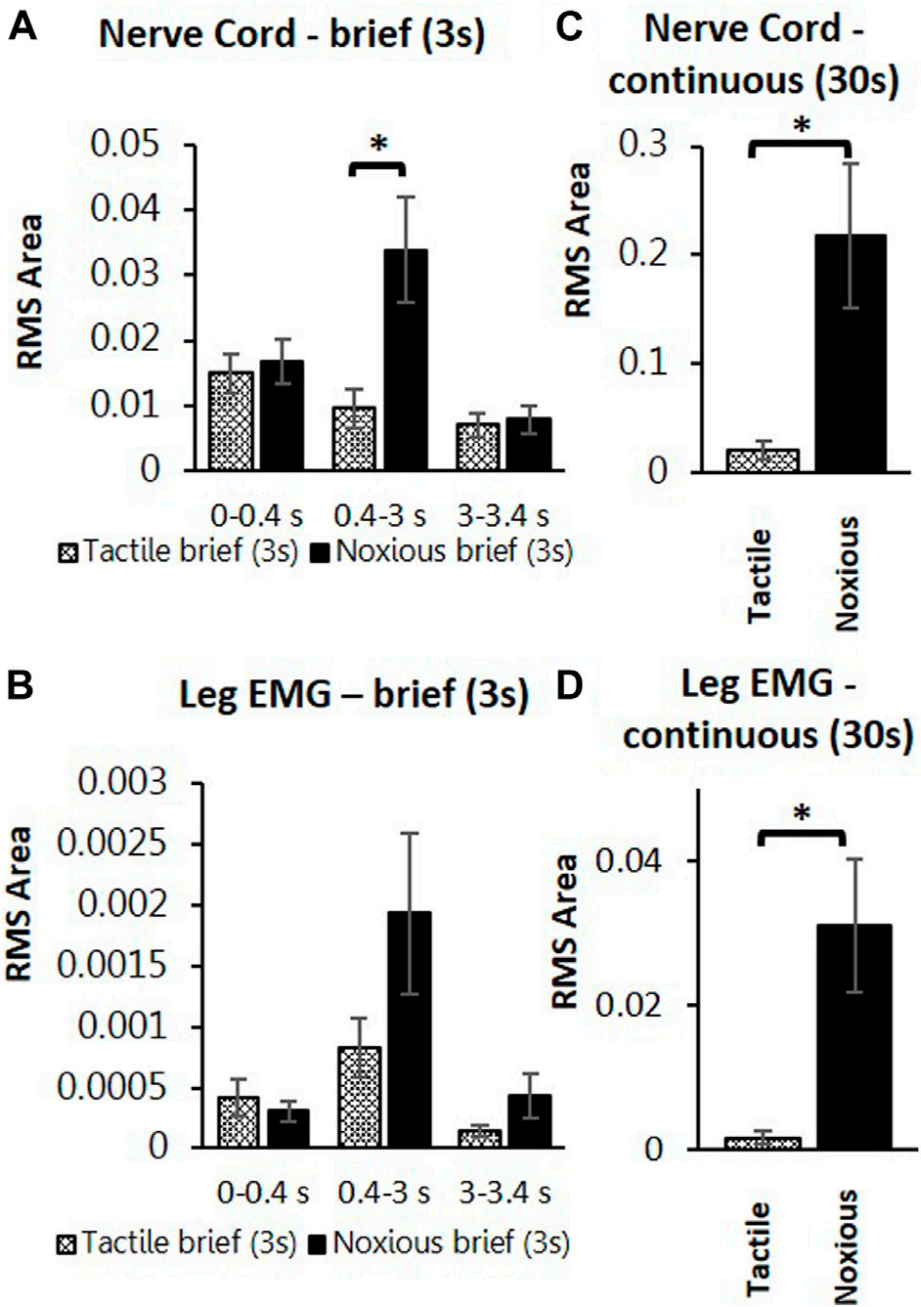
Continuous noxious stimulus evokes robust response in interneurons and motoneurons compared with tactile stimulus in stung cockroaches. **(A)** Averaged RMS area of the nerve cord response to brief tactile (White bar with cross) and noxious stimuli (Black bar), according to the time divisions as represented with grey lines inFigure 2A. Panel **(B)** is the same as panel **(A)** for the leg EMG response. **(C)** Averaged RMS area of nerve cord response to a continuous transition between tactile (White bar with cross) and noxious (Black bar) stimuli. Panel **(D)** is the same as panel **(C)** for the leg EMG response. Bars represent means ± SEM, significance is indicated with an asterisk.

To search for neuronal correlates of nocifensive behavior impairment in stung cockroaches, we performed a series of electrophysiological recordings from nociceptive interneurons projecting to the central complex (the location of the wasp sting). Recordings in stung cockroaches show that tactile and noxious heat stimuli elicit distinctive responses from postsynaptic, intersegmental projection interneurons (Figures 2, 3). Following a brief (3s) tactile stimulus (Figure 2A, n = 9), large amplitude spikes are evoked at the beginning and end of the stimulus. In contrast, responses to brief noxious stimuli (3s) consist of large amplitude spike responses at stimulus onset, followed by smaller amplitude spikes throughout the stimulus (Figures 2A,B). Both brief tactile and brief noxious stimuli recruit the slow coxal depressor motoneuron (Ds) but fail to engage the fast coxal depressor motoneuron (Df) (Figures 2A,B). Since brief noxious stimuli include both tactile and noxious components (Figures 2A,B), we applied a “continuous stimulus” to measure the pure nociceptive component of projection interneuron responses (Figures 2C,D). Responses to a continuous stimulus between tactile and noxious stimuli starts with a transient response to the tactile stimulus and an ongoing response to the noxious stimulus (Figure 2C, n = 7). In addition to the ongoing recruitment of Ds by the tactile component of the stimulus, the noxious component evokes a robust discharge of Df (Figures 2C,D). Figures 3A,B display quantification of responses shown in Figure 2A as measured by RMS area. The response is expressed in three time-bins corresponding to onset, duration, and termination of the stimulus (0–0.4, 0.4–3, and 3–3.4 s; vertical lines corresponding to this division are shown in Figure 2A). A significant increase in response to the noxious stimulus is found in the 0.4–3s time bin of the stimulus for the interneuron response (Figure 3A; *p* < 0.05, paired *t*-test). This is the time bin during which nociceptive fibers are recruited and mostly active, unlike the tactile fibers, which are firing at a rate close to their baseline-firing rate [see (Emanuel and Libersat, 2019)]. In the leg EMG response, no significant difference is found in any of the time bins (Figure 3B, paired *t*-test), although a trend of increase in the 0.4–3s time bin of the stimulus is evident, which parallels the increase in firing of nociceptive neurons. Figures 3C,D show RMS area differences in responses of the nerve cord and leg EMGs, respectively, to continuous transition stimuli. A significant increase in response to the noxious stimulus is found for the nerve cord response (Figure 3C, *p* < 0.05, paired *t*-test) and for the leg EMG response (Figure 3D, *p* < 0.05, Wilcoxon signed-rank test).

**FIGURE 4:**
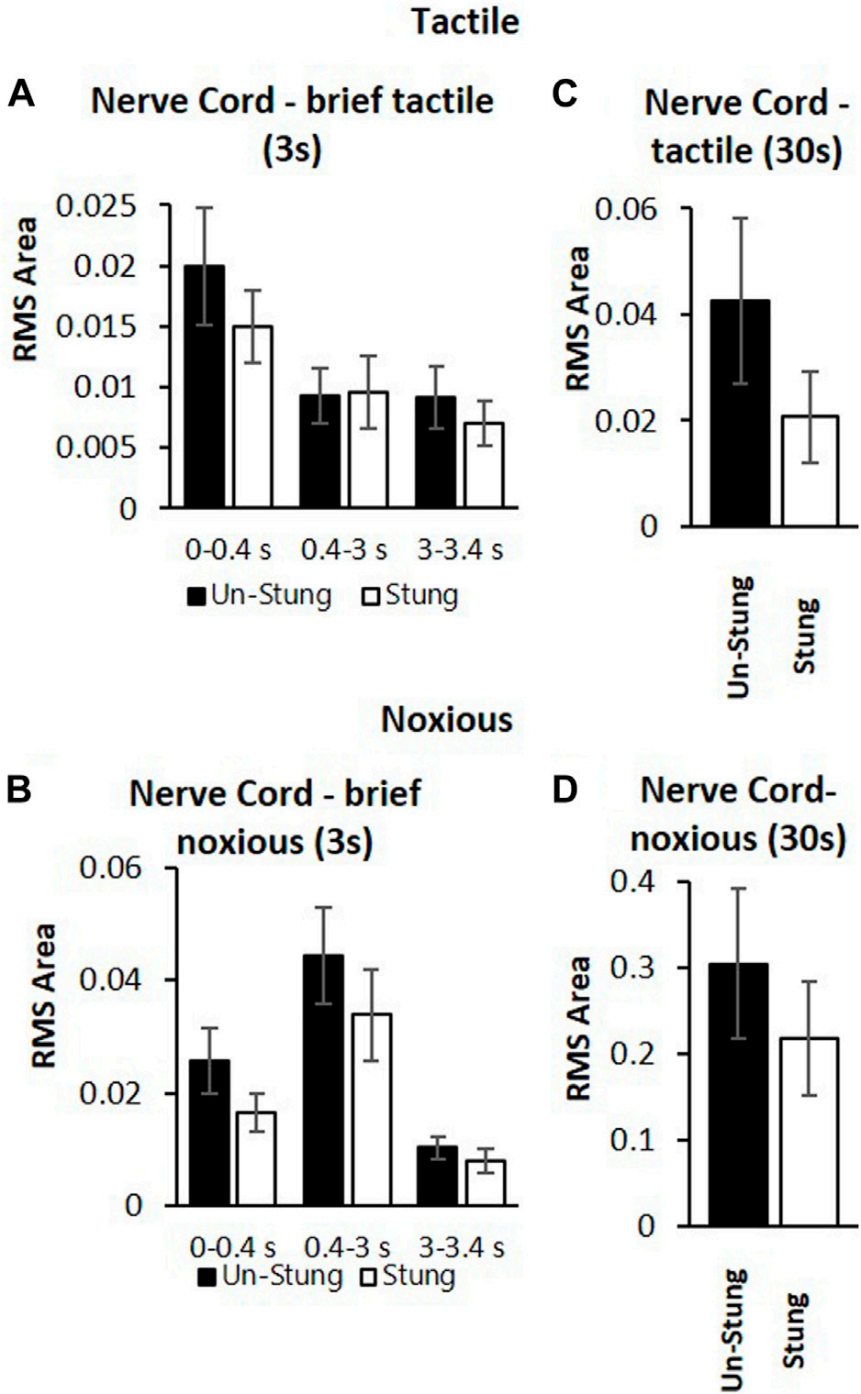
Post-synaptic inter-neurons in the nerve cord of stung (white bars) and un-stung (black bars) cockroaches are similarly active in response to both tactile stimuli and noxious stimuli. **(A)** Averaged RMS area of nerve cord response of un-stung (n = 20) and stung cockroaches (n = 9) to brief (3s) tactile stimuli. Panel **(B)** Averaged RMS area of the nerve cord response of un-stung (n = 20) and stung cockroaches (n = 9) to brief (3s) noxious stimuli. Panel **(C)** Averaged RMS area of the nerve cord response of un-stung (n = 13) and stung cockroaches (n = 7) to 30s tactile stimuli. Panel **(D)** Averaged RMS area of the nerve cord response of un-stung (n = 13) and stung (n = 7) cockroaches to 30 s noxious stimuli. For all panels, bars represent means ± SEM; significance is indicated with an asterisk (no significance was found).

These measurements are compared to those collected from un-stung cockroaches (Figure 4). Both groups of animals were subjected to the same experimental procedure and protocol. The stung group was exposed to a wasp sting 4–6 h prior to the beginning of the experiment. In this comparison, we first examine postsynaptic interneuron responses to a brief tactile and brief noxious stimulus lasting 3 s (Figures 4A,B) and after to continuous tactile stimulus, as well as a continuous noxious stimulus lasting 30 s (Figures 4C,D). When comparing nerve cord responses of stung and un-stung cockroaches to brief tactile (3 s) and 30 s tactile stimuli, no significant difference is found (Figures 4A–C; Mann-Whitney rank-sum test; the number of animals per group “n” is indicated in the figure legend). Likewise, when comparing nerve cord responses of stung and un-stung cockroaches to brief (3 s) and 30 s noxious stimuli, no significant difference was found (Figures 4B–D; Mann-Whitney Rank Sum Test; number of animals per group “n” is indicated in the figure legend). From this comparison, we conclude that ascending interneuron responses to tactile and noxious stimuli are comparable in un-stung and stung cockroaches.

### 3.3 Failure of behavioral responses to noxious laser stimuli in stung cockroaches

**FIGURE 5:**
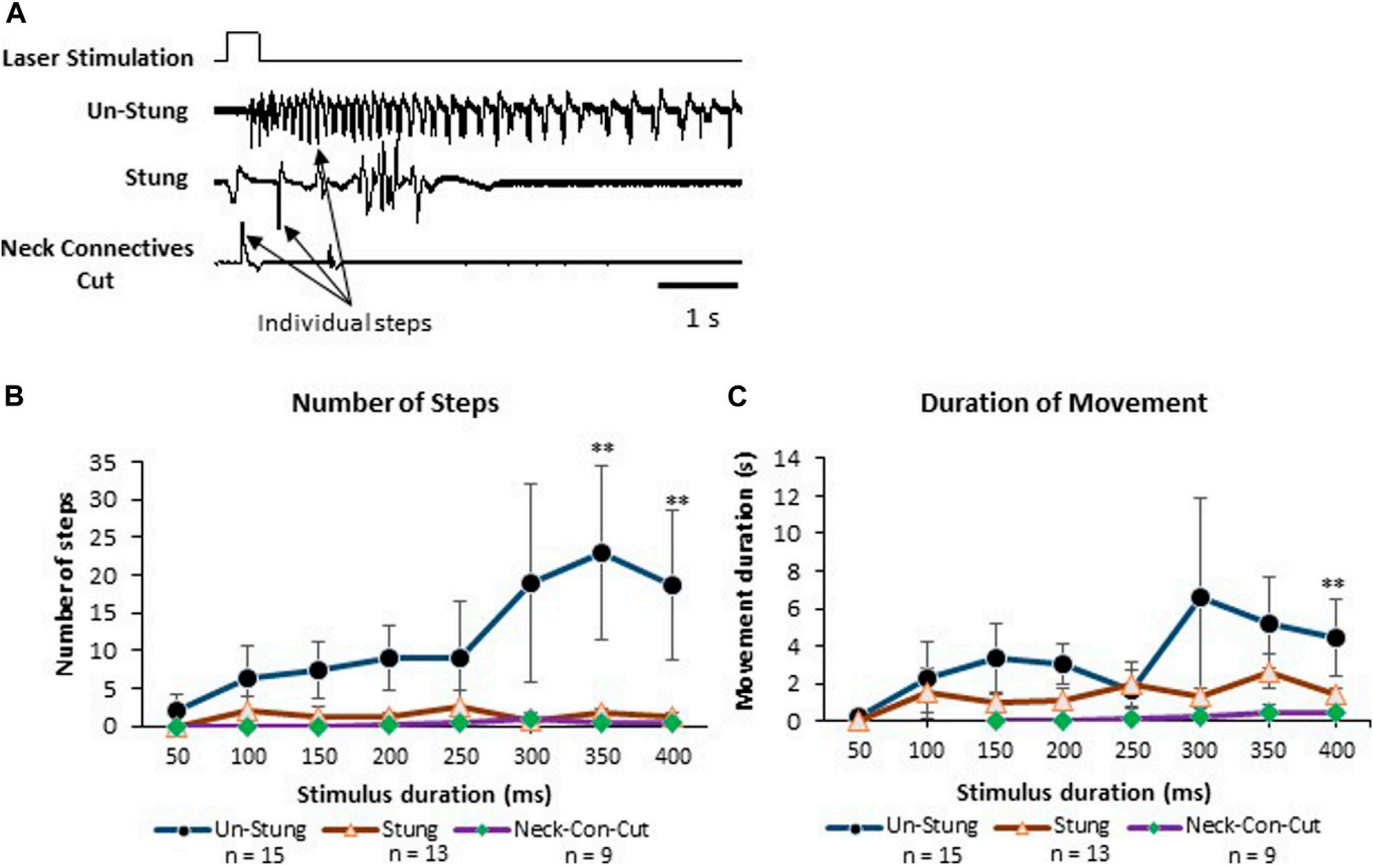
Stung and Neck Connectives Cut cockroaches fail to perform an escape response in response to a pure noxious stimulus. **(A)** cockroaches’ Leg movements (which represent walking or running) of Un-Stung (upper trace), Stung (middle trace) and Neck Connectives Cut (lower trace) in response to a 400 ms laser stimulus to the abdomen. Stimulus duration is indicated above the three traces. Individual steps are indicated by arrows. **(B, C)** Plots showing the Average ±SEM of the Number of Steps **(B)**, the Duration of Movement in seconds **(C)**. Significance is indicated with stars (*: *p* ≤ 0.05; **: *p* ≤ 0.01; ***: *p* ≤ 0.001). Significance is only shown where the un-stung group is significantly different from the two other groups.

In this set of experiments, behavioral responses to a pure noxious laser stimulus uncontaminated by the tactile component of the hot probe stimulus device were tested in tethered un-stung (n = 15), stung (n = 13) and Neck-Connectives Cut (n = 9) cockroaches (Figure 5). In the example depicted in Figure 5A, an un-stung cockroach responds to a 400 ms laser stimulus with a fast, long-lasting escape response. Stung and Neck Connective Cut cockroaches show a brief startle motor response to the same noxious stimulus, but fail to engage an escape response. Figure 5 shows the number of steps (B) and duration of movement (C) for laser stimulus durations ranging from 50 to 400 ms. From these experiments, we found that the number of steps and duration of the escape response were both significantly higher in response to laser stimuli exceeding 300 ms in unstung cockroaches compared with stung or connectives-cut cockroaches (Figure 5B). Thus, in control, unstung cockroaches, a laser stimulus of 400 ms triggers a robust escape response, whereas the same stimulus fails to initiate a full escape behavior for stung and Neck Connective Cut cockroaches.

### 3.4 The central complex detects nociceptive input

**FIGURE 6:**
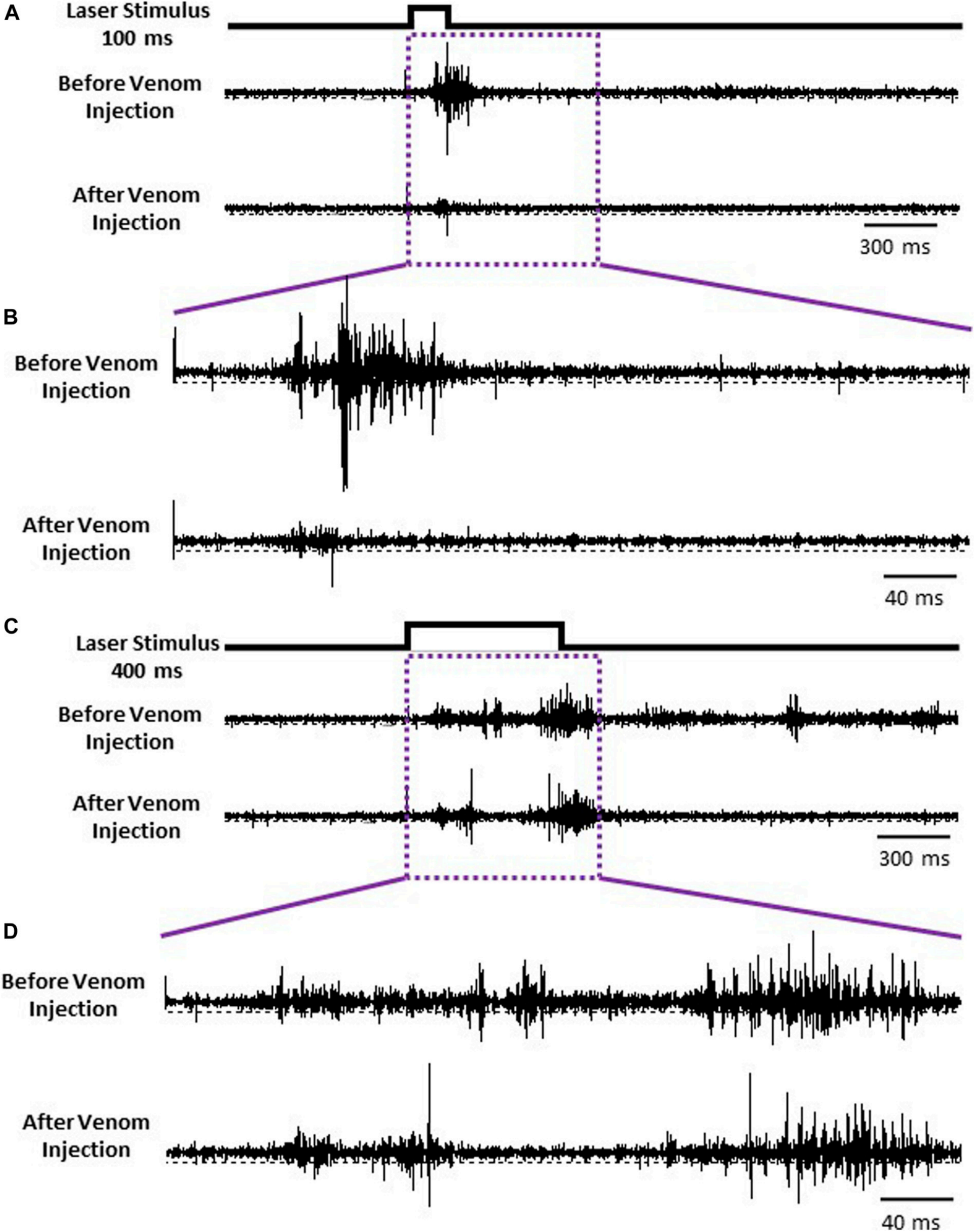
CX is the final center for processing nociceptive input. **(A)** Representative examples recordings of CX for ineffective 100 ms laser stimulus before (Upper trace) and after the venom injection (Lower trace). **(B)** Enlarged view of traces in purple box in panel **(A)**. **(C)** Representative examples recordings of CX for an effective 400 ms laser stimulus before and after the venom injection. **(D)** Enlarged view of traces in purple box in panel **(C)**.

Given that nociceptive projection interneurons transfer information to the head ganglia in stung cockroaches [see also Emanuel and Libersat (2019)], we hypothesized that processing of this input by the central complex (CX) might be modulated in stung cockroaches. As a first step in this analysis, we compared neuronal responses in the CX following an ineffective laser stimulus (100 ms) evoking no nocifensive escape response to an effective laser stimulus (400 ms) that evokes a full nocifensive escape response in intact animals before and after venom injection in otherwise tethered animals (Figure 6). We analyzed five different parameters of CX responses encompassing 11 individually identified units from 7 animals: 1) latency, 2) peak firing rate, 3) total evoked activity, 4) evoked average firing rate, and 5) response duration to either type of stimulus.

#### 3.4.1 Latency and peak firing rate of CX neurons do not correlate with nocifensive behavioral threshold

**FIGURE 7:**
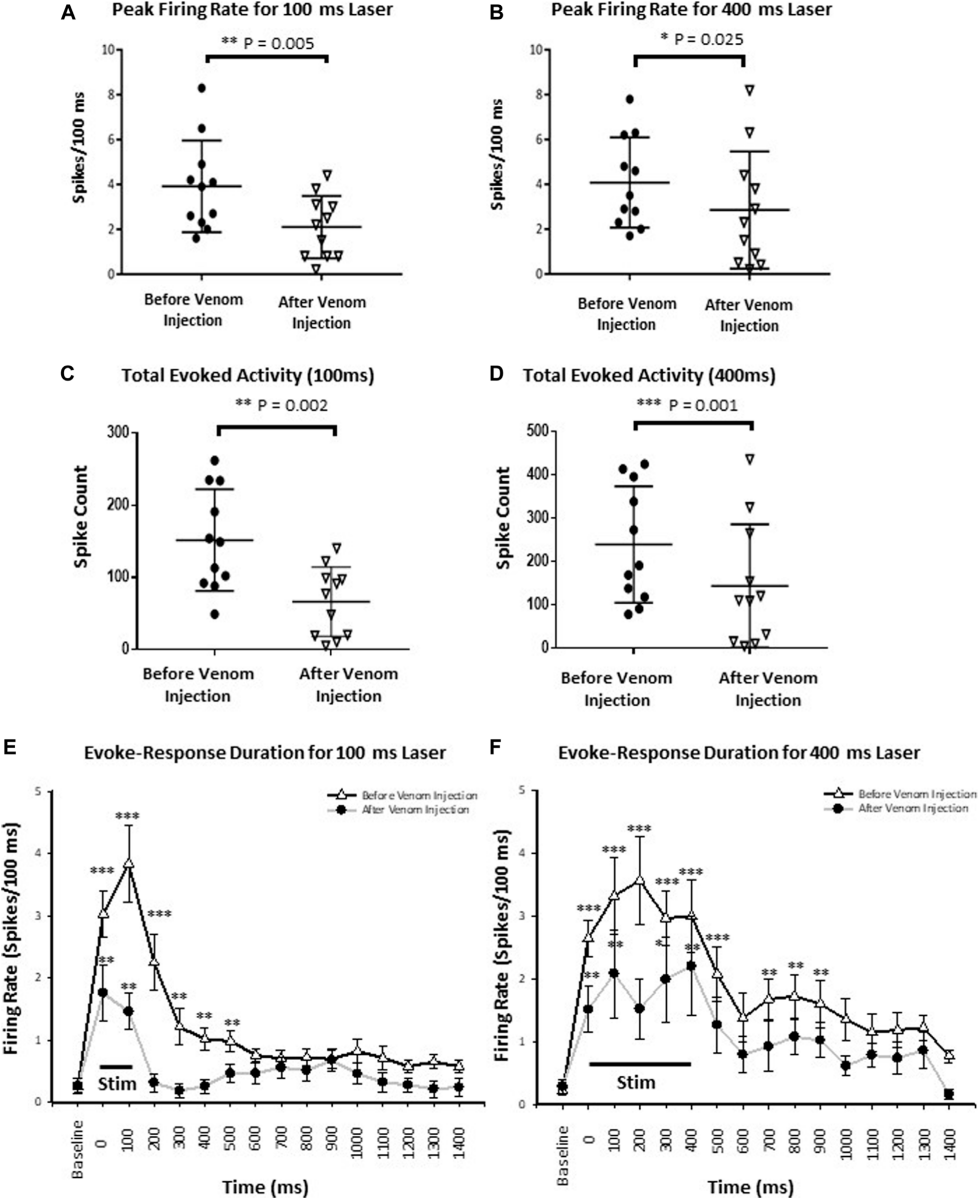
Wasp venom reduces the CX activity in response to nociceptive stimuli. **(A)** Peak firing rate decreases after venom injection for ineffective stimuli (100 ms laser stimuli). **(B)** Same as panel **(A)** for effective stimuli (400 ms laser stimuli). **(C)** Total Evoked activity decreases after venom injection for ineffective stimuli (100 ms laser). **(D)** Same as panel **(C)** for effective stimuli (400 ms laser stimuli). Black circles represents data point before the venom injection for ineffective and effective stimulus, downward triangles represents data point after venom injection. **(E)** Peri-stimulus time histograms for ineffective 100 ms laser stimulus before (black line with white triangle) and after venom injection (grey line with black dot). The black line at the bottom represents the duration of stimulus. The mean response and duration of response decreases after venom injection for each stimulus type. **(F)** Same as panel **(E)** for effective stimulus. For all panels, points represent means ± SEM, significance is indicated with an asterisk (*: *p* ≤ 0.05; **: *p* ≤ 0.01; ***: *p* ≤ 0.001, n = 11 units from 7 animals).

To assess speed of information processing as well as recruitment of additional neuronal processes by an effective stimulus (400 ms) compared to the ineffective stimulus (100 ms), we quantified stimulus response latency and peak firing rate, respectively. Stimulus-response latency was calculated as the time between onset of the stimulus and peak neuronal response, defined as the maximum number of spikes within a 100 ms time bin from onset of the stimulus. For an ineffective stimulus, stimulus response latency was the same before and after venom injection (*p*-value = 0.341). Likewise, for the effective stimulus, stimulus response latency also remained the same before and after venom injection (*p*-value = 0.829). In contrast, peak firing rate drops significantly after venom injection for an ineffective stimulus (Figure 7A, mean ± SEM: 3.918 ± 0.617 before venom injection compared with 2.045 ± 0.413 after venom injection, *p*-value = 0.005). Similarly, the peak firing rate also decreases significantly after venom injection for an effective stimulus (Figure 7B, Mean ± SEM: 4.082 ± 0.607 before venom injection, 2.855 ± 0.788 after venom injection, *p*-value = 0.025). Thus, although peak firing rate decreases after venom injection, this alone cannot account for nocifensive behavior failure in unstung cockroaches.

#### 3.4.2 Total evoked activity and evoked average firing rate in CX neurons decrease following venom injection

We defined the total evoked activity as the number of spikes for 1 s after the stimulus onset (Supplementary Table S3). Here, we found, for the ineffective stimulus, the total evoked activity drops significantly after venom injection (Figure 7C, Mean ± SEM, *p*-value shown in Supplementary Table S3). Similarly, total evoked activity is also reduced after venom injection for effective stimuli (Figure 7D, Mean ± SEM, *p*-value shown in Supplementary Table S3).

We performed a more detailed analysis of this reduction in total evoked activity by comparing average evoked firing rate responses to noxious stimuli in the CX under key experimental conditions: 1. An ineffective stimulus applied before and after venom injection, 2. An effective stimulus applied before and after venom injection. Evoked average firing rate was calculated in 250 ms time bins from 500 ms before to 1.5 s after onset of the laser stimulus (Supplementary Figure S1).

For all stimuli, evoked average firing rate in the CX decreases significantly after venom injection (Supplementary Figure S1). Furthermore, the level of significance decreases with respect to time from the onset of the stimulus. For an ineffective stimulus evaluated during a 750 ms time window from stimulus onset, evoked average firing rate differs significantly before and after venom injection (Supplementary Figure S1, mean ± SEM, *p*-value shown in Supplementary Table S1). Likewise, for the effective stimulus, evoked average firing rate before and after venom injection differs significantly within the 1,250 ms time window from stimulus onset (Supplementary Figure S1, mean ± SEM, *p*-value shown in Supplementary Table S1). Significance is indicated with asterisks for ineffective stimuli and with diesis for effective stimuli (^*/‡^: *p* ≤ 0.05; ^**/‡‡^: *p* ≤ 0.01; ^***/‡‡‡^: *p* ≤ 0.001, n = 11 units from 7 animals).

In summary, we conclude that before venom injection, total evoked activity in the CX increases in proportion to increased stimulus duration. Furthermore, both ineffective and effective stimuli evoke an increase in average firing rate immediately after stimulus onset (Mean average firing rate ±SEM: 3.269 ± 0.466, 3.204 ± 0.484 respectively for ineffective and effective stimuli). However, effective stimuli result in longer duration activity as compared to ineffective stimuli. Following venom injection, total evoked activity is reduced for both ineffective and effective stimuli. This diminished activity results from a shortening of the response window for effective stimuli. Thus, responses triggered by effective stimuli after venom injection are reduced.

#### 3.4.3 Duration of CX responses decreases following venom injection

The aforementionned results show that neither total evoked activity nor peak firing rate are correlated with behavioral results obtained from unstung and stung cockroaches exposed to noxious laser stimuli. Therefore, in searching for a parameter correlated with the behavioral data, we analyzed CX response duration by sampling the response of individual CX units for every 100 ms bin. Each binned response is then compared to baseline firing before stimulus onset. Thus, significance is only shown where the laser evoked neuronal activity is significantly different from pre-stimulus baseline (spontaneous) activity. Before venom injection, response duration varies with the duration of the stimulus (100 vs. 400 ms) and decreases after venom injection for either type of stimulus. Ineffective stimuli evoke a 600 ms response duration before venom injection, which is reduced to 200 ms after venom injection (Figure 7E, mean ± SEM and *p*-value in Supplementary Table S4). Effective stimuli evoke a response duration of 1 s before venom injection (though no significant difference was found for 600–700 ms bins, *p*-value = 0.275), which is reduced to 500 ms after venom injection (Figure 7F, mean ± SEM and *p*-value in Supplementary Table S4). Significance is indicated with stars (*: *p* ≤ 0.05; **: *p* ≤ 0.01; ***: *p* ≤ 0.001). Hence, duration of CX neuron firing in response to noxious stimuli is likely correlated with escape responses and could be the causal link.

#### 3.4.4 Wasp venom causes failure of nocifensive behavior

**FIGURE 8:**
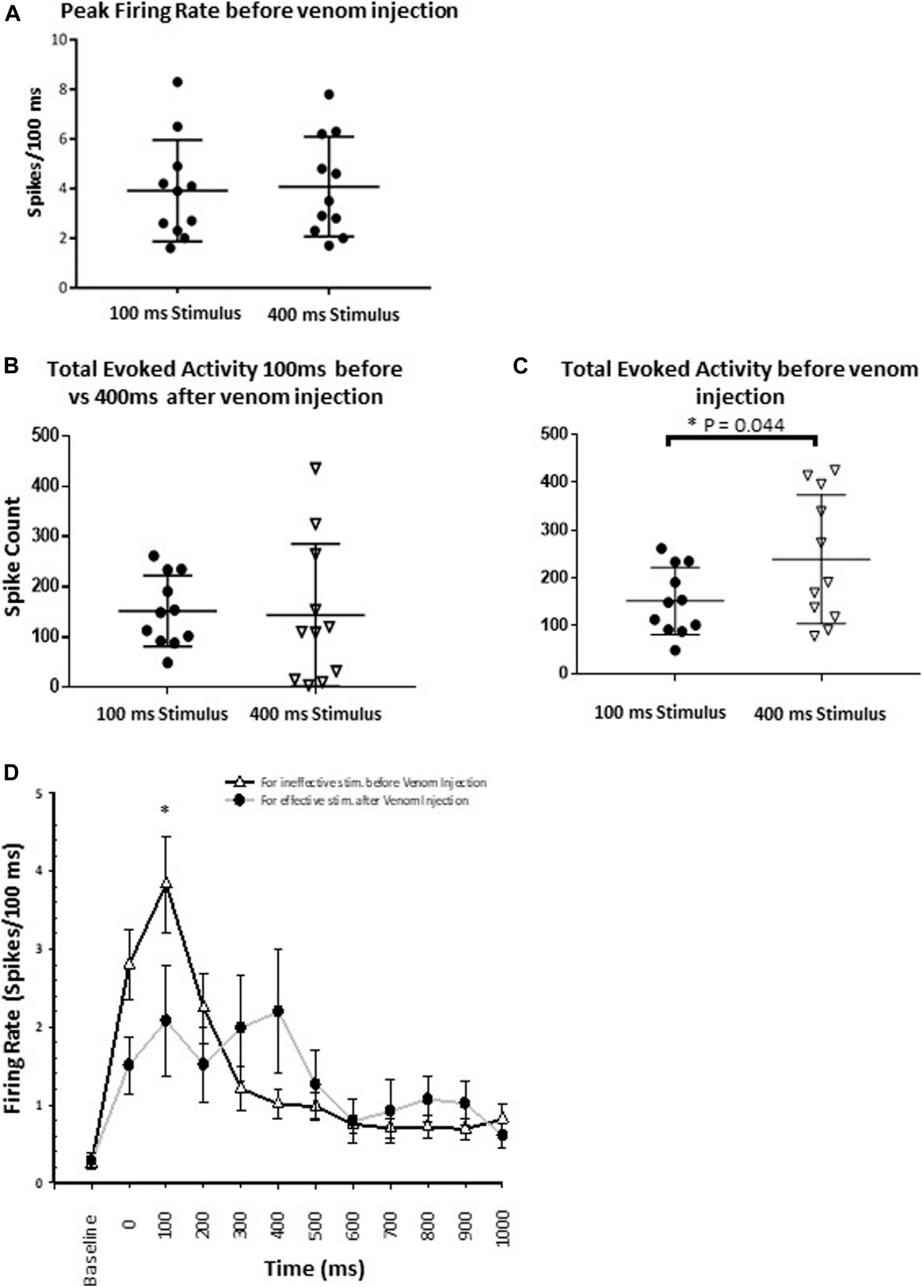
Wasp venom causes the desensitization of nociceptive response in CX. **(A)** Comparison of Peak firing rate before the venom injection shows no difference in peak firing rate for either type of stimuli (Ineffective and effective). This shows that no additional neuronal processes are recruited by effective stimuli. **(B)** For ineffective stimuli (100 ms) total evoked activity before the venom injection remains to be similar to the total evoked activity for effective stimulus (400 ms) after the venom injection. **(C)** Comparison of total evoked activity before the venom injection for either type of stimuli. Effective stimuli (400 ms laser stimuli) triggers significantly higher activity in the CX. **(D)** Peri-stimulus time histograms (PSTH) for ineffective 100 ms laser stimulus before the venom injection (black line with white triangle) and for effective 400 ms laser stimulus after venom injection (grey line with black dot). PSTH for ineffective stimuli before the venom injection is similar to the PSTH for effective stimuli after venom injection. Wasp venom increases the threshold for nociceptive response in CX. points represent means ± SEM, significance is indicated with an asterisk (*: *p* ≤ 0.05; **: *p* ≤ 0.01; ***: *p* ≤ 0.001, n = 11 units from 7 animals).

Our results provide evidence that wasp venom reduces the CX response to nociceptive stimuli and establish the correlation between CX neuronal activity and behavioral data. Before venom injection, peak firing rate in response to both ineffective and effective stimuli are similar (Figure 8A, *p*-value = 0.777) and cannot account for the behavioral change in nocifensive threshold. Moreover, behavioral experiments indicate that the escape response occurs only for effective stimuli (350–400 ms, Figure 5) in un-stung cockroaches. Thus, to estimate differences in CX activity for effective (400 ms stimulus) vs. ineffective stimuli (100 ms stimulus), we compared total evoked activity resulting from either type of stimulus before venom injection. Here we found that total evoked activity for effective stimuli before the venom injection is significantly higher (Figure 8C, *p*-value = 0.044).

We also compared CX responses to an effective stimulus after venom injection to those following an ineffective stimulus before venom injection, as no escape response occurs under either condition. In this comparison, for ineffective stimuli, total evoked activity before the venom injection is similar to total evoked activity for effective stimuli after the venom injection (Figure 8B, for ineffective stimuli: Mean spike count ±SEM: 151.73 ± 21.23, for effective stimuli: Mean spike count ±SEM: 143.64 ± 42.90, *p*-value = 0.850). Likewise, neuronal activity within the response duration (1 s) is also assessed for these two conditions (Figure 8D). No significant difference in the neuronal activity is found, except for the 100–200 ms after the stimulus onset (Figure 8D, *p*-value = 0.033). Within this time window, ineffective stimuli before venom injection trigger significantly higher evoked activity as compared to effective stimuli after venom injection (Figure 8D, mean ± SEM: for ineffective stimulus 3.836 ± 0.617, for effective stimulus 2.082 ± 0.703).

## 4 Discussion

In the present study, we demonstrated that envenomation of the cockroach brain by the jewel wasp impairs nocifensive behavior (Figures 1, 5). Such impairment occurs only after nociceptive input reaches the brain, where activity of the fan-shaped body in the CX is altered in ways that help to explain loss of nocifensive behavioral responses.

Projection interneurons of both un-stung and stung cockroaches respond similarly to either tactile or nociceptive information (Figures 2–4). Responses to noxious stimuli (brief or 30 s continuous stimuli) exceed responses to tactile stimuli (Figures 3A–C), just as was observed in un-stung cockroaches (Emanuel and Libersat, 2019). Thus, although similar nociceptive information is carried forward to the brain and thoracic locomotory centers of both un-stung and stung cockroaches, the latter cannot execute an enhanced motor response to brief noxious stimuli as compared to brief tactile stimuli (Figure 3B). However, stung cockroaches do show an elevated motor response threshold to continuous (30 s) noxious stimuli as compared to tactile stimuli (Figure 3D). This is consistent with previous studies showing that stung cockroaches have an elevated threshold to electric shock stimuli (Gavra and Libersat, 2010).

Our findings are consistent with previous work showing that responses of wind sensitive interneurons are unaffected in stung cockroaches (Fouad et al., 1994). This is to be expected, since inability of stung cockroaches to engage in escape behavior regardless of the stimulus type occurs only when the wasp envenomates the head ganglia (Libersat et al., 2009). In the present study, brief or 30 s continuous tactile stimuli did not induce a robust motor response in either un-stung or stung cockroaches (data not shown), likely attributable to rapid adaptation exhibited by tactile-responsive interneurons (Emanuel and Libersat, 2019). Moreover, tactile-evoked motor responses do not increase with increasing stimulus duration (Ritzmann and Pollack, 1994). Finally, and yet importantly, it is well established that the cockroach motor responses to wind or tactile stimuli are drastically reduced in dissected or restrained animals (Ritzmann, 1981). This presumably accounts for differences in evoked motor responses in restrained/dissected and intact (Figure 1B) stung and un-stung cockroaches to tactile stimuli. Likewise, such a discrepancy appears between behavioral responses of intact vs. dissected stung cockroaches to brief tactile and noxious stimuli (Emanuel and Libersat, 2019). The robust motor response of un-stung cockroaches to brief noxious stimuli is probably due to persistent activity of nociceptive postsynaptic interneurons throughout the duration of the noxious stimulus (Emanuel and Libersat, 2019). Ongoing activity of nociceptive postsynaptic interneurons in stung cockroaches (Figure 2; Figures 3A–C) evokes a weaker motor response to brief noxious stimuli in stung cockroaches as compared to un-stung cockroaches (Figure 4B). This is likely caused by a modulatory effect of the wasp venom on higher centers in cockroach head ganglia, which regulate thoracic motor centers (Libersat and Gal, 2013; Emanuel et al., 2020). Reduction in descending tonic and permissive activity to thoracic ganglia and along the nerve cord could be responsible for a diminution in the behavioral and muscular response of stung cockroaches to brief noxious stimuli applied to the abdomen as compared to un-stung cockroaches. Stung cockroaches do show an elevated motor response to continuous (30 s) noxious stimuli as compared to tactile stimuli (Figure 3D). This might reduce differences between leg muscle responses of un-stung and stung cockroaches to continuous (30 s) noxious stimuli.

Use of a laser as a pure nociceptive stimulus (Mukherjee et al., 2020) led to fast, long-lasting escape responses in control un-stung animals, whereas stung cockroaches fail to respond (Figure 5). In this set of experiments, we added a group of cockroaches with severed neck connectives, eliminating communication between head ganglia with thoracic circuitries, the rationale being that descending commands from the head ganglia are crucial for the full escape behavior (Schaefer and Ritzmann, 2001). Interestingly, even in the absence of head ganglia, ascending interneurons in the nerve cord are still able to recruit thoracic motor circuitries to evoke a startle or “local” reflex (Figure 5A). A similar phenomenon was reported by Horridge (1962), who observed that headless cockroaches respond to an electrical shock by raising their legs. Similarly, while both stung and neck connective cut cockroaches do not show a full escape response to a noxious stimulus, they still respond with a startle response that is mediated by thorax motor circuitries. For comparison, this could be related to the nociceptive spinal local reflexes in mammals, which operate in the absence of a brain. Our results show that a laser stimulus of 400 ms duration applied to unstung cockroaches initiates an escape response consisting of a higher number of steps for longer duration compared to stung and neck-connective cut animals (Figure 5B). Thus, a laser stimulus of 400 ms is considered an effective stimulus or the stimulus threshold for evoking nocifensive behavior. These results are again consistent with the fact that stung cockroaches have an elevated behavioral threshold to nociceptive stimuli (Gavra and Libersat, 2010). In addition, they also indicate the importance of head ganglia to initiate a full escape response.

Within the head ganglia, the CX is of principal interest due to its well-established role in initiation and maintenance of walking (Guo et al., 2014; Kaiser and Libersat, 2015). We previously showed using radio-labelled wasps that the fan-shaped body of the CX is a primary target for the sting (Haspel et al., 2003). A recent study of nociceptive behavior in *Drosophila* demonstrated that activation of neurons in the fan-shaped body, a subregion of the CX, leads to onset of nocifensive behavior (Hu et al., 2018). Thus, it is reasonable to hypothesize that absence or diminished nocifensive behavior in stung cockroaches is a consequence of reduced CX activity in response to a nociceptive input. Given these facts, all recordings presented in this study were performed in the fan-shaped body of the CX.

To investigate how wasp venom affects CX neuronal activity to nociceptive input, we compared its response to an effective laser stimulus (400 ms) with that of an ineffective laser stimulus (100 ms) before and after the venom injection (Figure 6). Our results show that envenomation suppresses the CX neuronal response, as peak firing rate declines significantly after venom injection for either type of stimuli (Figures 7A,B). However, venom injection into the CX does not affect the speed of nociceptive information transfer, as the latency of neuronal response is the same before and after venom injection for either type of stimuli.

To further characterize the CX response to noxious stimuli, we estimated total evoked activity and evoked average firing rate before and after the venom injection. Our results show that, regardless of stimulus type (effective or ineffective), total evoked CX activity is reduced significantly after venom injection (Figures 7C,D). Likewise, by analyzing evoked average firing rate, we found that injection of wasp venom into the CX significantly reduces evoked average firing rate for both the stimulus types (Supplementary Figure S1, red bars with horizontal line for 100 ms laser stimulus, grey bars with cross for 400 ms stimulus). Thus, the intensity of CX response is diminished as all three parameters—peak firing rate, total evoked activity and evoked average firing rate—are reduced after venom injection. This is not the first instance where a reduction in neuronal activity due to wasp envenomation has been reported. Previous studies showed a similar reduction in neuronal activity of the gnathal ganglion (the other targeted head ganglion by the wasp sting) in stung cockroaches as compared to un-stung cockroaches (Gal and Libersat, 2010). This trend of reduction is also evident in the duration of CX response. Our result shows that an ineffective laser stimulus elevates CX activity for a much shorter time (600 ms, Figure 7E) as compared to an effective stimulus (1,000 ms, Figure 7F). The duration of activity further declines after venom injection. This limits the neuronal response duration up to 500 and 200 ms for effective and ineffective stimuli, respectively (Figures 7E,F). Hence, our results provide substantial evidence that wasp venom reduces CX responses to nociceptive input.

Our behavioral experiments indicate that the escape response occurs only for effective stimuli (350–400 ms, Figure 5B) in un-stung cockroaches. Thus, to find a correlate between our behavioral results and the venom induced modulation of neuronal activity in the CX, we compared the total evoked activity resulting from either type of stimulus before venom injection. In this comparison, the total evoked activity triggered by an effective stimulus is significantly higher (Figure 8C).

Furthermore, we compared total evoked activity for effective stimuli after venom injection to total evoked activity for ineffective stimuli before venom injection, as no escape response was triggered in either of the conditions (Figure 8B). In this comparison, total evoked activity is similar for both conditions (Figure 8B). In addition, we found that within the response duration window (1,000 ms), neuronal activity triggered by an effective stimulus after venom injection is comparable to neuronal activity triggered by an ineffective stimulus before venom injection (Figure 8D). For these two conditions, neuronal activity differs only for the 100–200 ms time bin after the onset of stimulus (Figure 8D, *p*-value = 0.033). Within this time window, ineffective stimuli before venom injection trigger significantly higher evoked activity as compared to effective stimuli after venom injection.

Given these facts, we revealed the best correlated CX activity parameter with the behavioral results. A brief noxious stimulus (100 ms) and a longer duration noxious stimulus (400 ms) both fail to induce escape behavior in un-stung cockroaches and stung cockroaches respectively. This correlates with both the duration and total spiking evoked response of CX neurons, which does not differ for a 100 ms stimulus before venom injection compared with a 400 ms stimulus after venom injection.

Numerous investigations suggest that the CX is involved in sensory integration and pre-motor processing (Pfeiffer and Homberg, 2014) but is also involved in ongoing regulation of locomotion and other motor behaviors (Strauss and Heisenberg, 1993; Martin et al., 1999; Bender et al., 2010). For instance, injection of acetylcholine or direct electrical stimulation of the CX initiates singing in insects (Bhavsar et al., 2017). But more relevant here in cockroaches, some CX units show increased firing rates preceding initiation of locomotion, while stimulation of the CX promotes walking. These observations implicate the CX as a command center for walking (Guo and Ritzmann, 2013). Furthermore, neuropeptides in the CX modulate locomotor behavior in *Drosophila* (Kahsai et al., 2010).

Venom-induced suppression of aversive nociceptive and escape behaviors have been described for both parasitoid and predatory animals. One report of parasitic manipulation of nociceptive response in the host involves mice infected with *Eimeria vermiformis*. Infected mice display increases in centrally mediated antinociceptive responses, apparently *via* naloxone-sensitive opioid signaling (Kavaliers and Colwell, 1993). Analgesic properties of venom components also are present in snake venom (Pu et al., 1995; Chen et al., 2006). Mixtures of toxins comprising a “nirvana cabal” in predatory net-hunting marine snails of the genus *Conus* suppress escape responses in fish *via* actions on adrenoreceptors, NMDA receptors, and *via* insulin-related peptides producing hypoglycemia (Olivera, 2002; Dutt et al., 2019). Of therapeutic relevance is ω-conotoxin MVIIA, a peptide from *Conus magus*, which when applied intrathecally produces analgesia in morphine-tolerant patients (Olivera et al., 1987). This peptide is known as Ziconotide (SNX-111; Prialt) (Pope and Deer, 2013; Pope et al., 2017).

The results presented in the current study reveal an additional function of the CX as a nociceptive processing center. In particular, our findings suggest that the fan-shaped body from which most of our recordings were acquired is involved in this processing. This is in agreement with a recent study demonstrating that fan-shaped body neurons in the *Drosophila* brain regulate both innate and conditioned nociceptive avoidance (Hu et al., 2018). We propose that the wasp uses an opiate receptor-binding molecule in its venom (Gavra and Libersat 2010; Kaiser et al., 2019) to suppress incoming nociceptive traffic, preventing the occurrence of nocifensive behavior in stung cockroaches. To the best of our knowledge, this is the first example of a venomous animal that modulates nociception center in its host/prey for the benefit of its progeny.

## Data Availability

The raw data supporting the conclusions of this article will be made available by the authors, without undue reservation.
