# Supplementary material for: Suppression of host nocifensive behavior by parasitoid wasp venom
Source: Front Physiol. 2022 Aug 12;13:907041. doi: 10.3389/fphys.2022.907041 (PMC9411936; doi:10.3389/fphys.2022.907041)
Supplement: Supplementary file 1 [file Presentation1.pptx]

## Slide 1
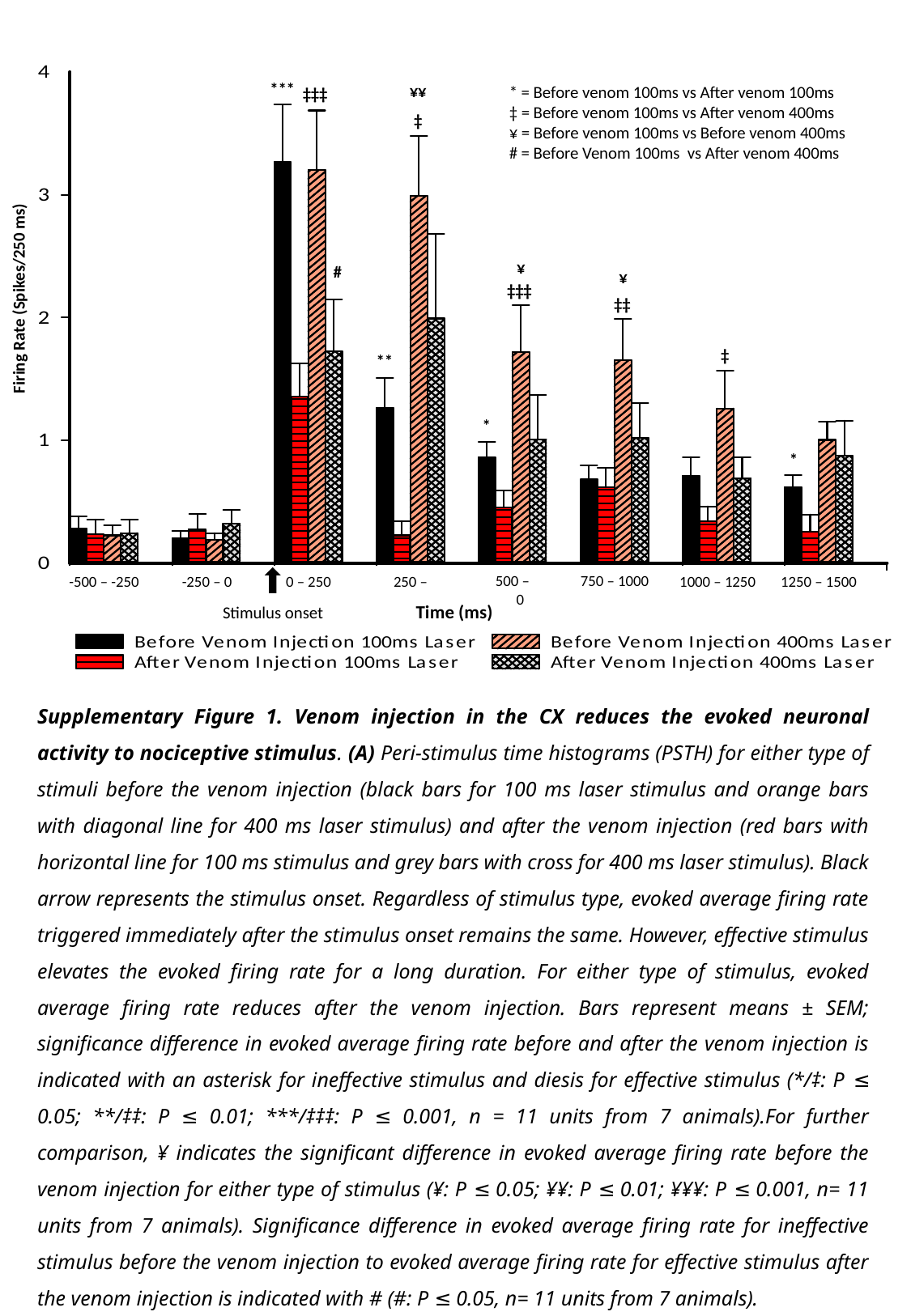

***
¥¥
* = Before venom 100ms vs After venom 100ms
‡ = Before venom 100ms vs After venom 400ms
¥ = Before venom 100ms vs Before venom 400ms
# = Before Venom 100ms vs After venom 400ms
‡‡‡
‡
¥
#
¥
‡‡‡
Firing Rate (Spikes/250 ms)
‡‡
‡
**
*
*
750 – 1000
500 – 750
0 – 250
250 – 500
-250 – 0
1000 – 1250
-500 – -250
1250 – 1500
Time (ms)
Stimulus onset
Supplementary Figure 1. Venom injection in the CX reduces the evoked neuronal activity to nociceptive stimulus. (A) Peri-stimulus time histograms (PSTH) for either type of stimuli before the venom injection (black bars for 100 ms laser stimulus and orange bars with diagonal line for 400 ms laser stimulus) and after the venom injection (red bars with horizontal line for 100 ms stimulus and grey bars with cross for 400 ms laser stimulus). Black arrow represents the stimulus onset. Regardless of stimulus type, evoked average firing rate triggered immediately after the stimulus onset remains the same. However, effective stimulus elevates the evoked firing rate for a long duration. For either type of stimulus, evoked average firing rate reduces after the venom injection. Bars represent means ± SEM; significance difference in evoked average firing rate before and after the venom injection is indicated with an asterisk for ineffective stimulus and diesis for effective stimulus (*/‡: P ≤ 0.05; **/‡‡: P ≤ 0.01; ***/‡‡‡: P ≤ 0.001, n = 11 units from 7 animals).For further comparison, ¥ indicates the significant difference in evoked average firing rate before the venom injection for either type of stimulus (¥: P ≤ 0.05; ¥¥: P ≤ 0.01; ¥¥¥: P ≤ 0.001, n= 11 units from 7 animals). Significance difference in evoked average firing rate for ineffective stimulus before the venom injection to evoked average firing rate for effective stimulus after the venom injection is indicated with # (#: P ≤ 0.05, n= 11 units from 7 animals).

## Slide 2
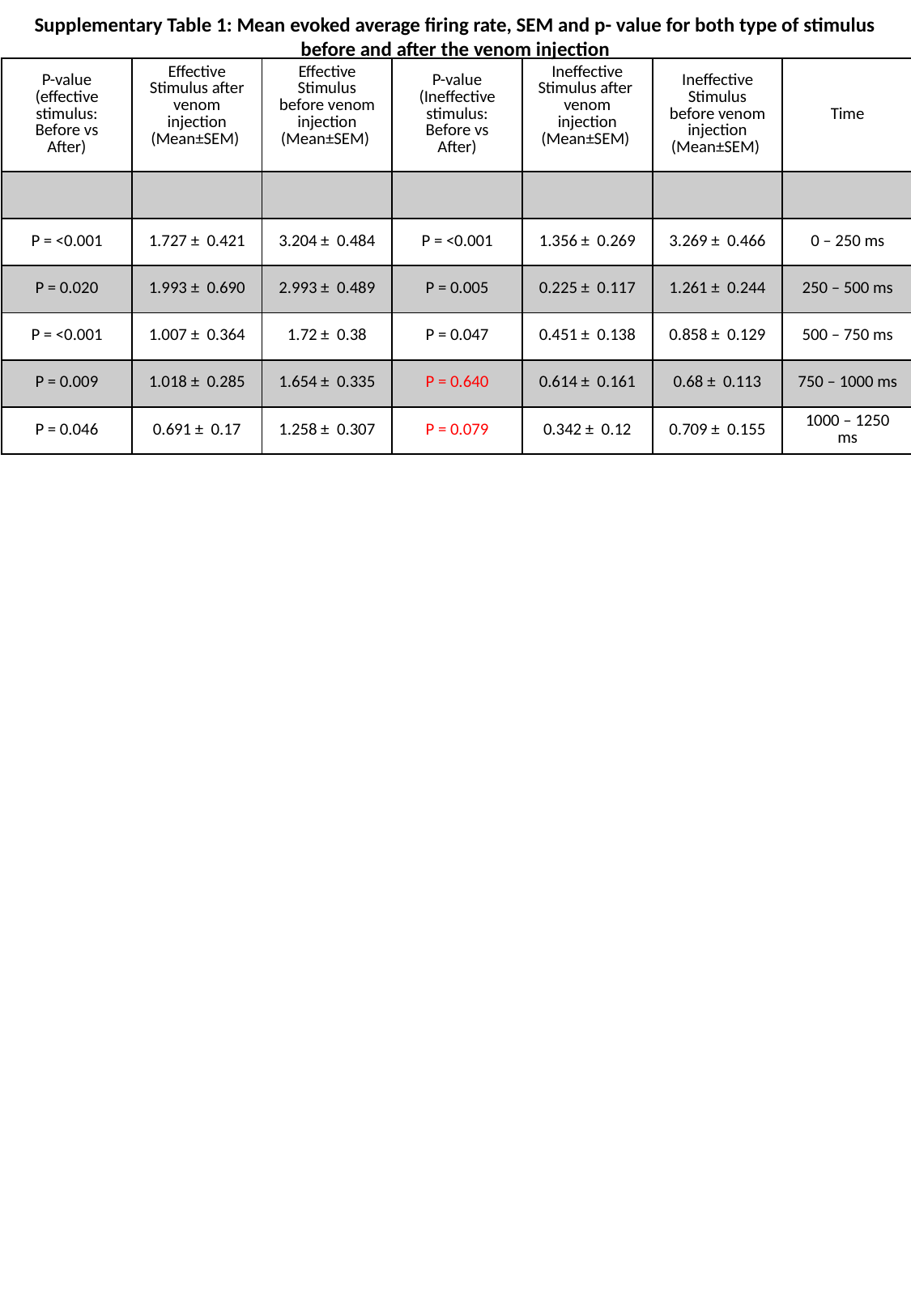

Supplementary Table 1: Mean evoked average firing rate, SEM and p- value for both type of stimulus before and after the venom injection
| P-value (effective stimulus: Before vs After) | Effective Stimulus after venom injection (Mean±SEM) | Effective Stimulus before venom injection (Mean±SEM) | P-value (Ineffective stimulus: Before vs After) | Ineffective Stimulus after venom injection (Mean±SEM) | Ineffective Stimulus before venom injection (Mean±SEM) | Time |
| --- | --- | --- | --- | --- | --- | --- |
| | | | | | | |
| P = <0.001 | 1.727 ± 0.421 | 3.204 ± 0.484 | P = <0.001 | 1.356 ± 0.269 | 3.269 ± 0.466 | 0 – 250 ms |
| P = 0.020 | 1.993 ± 0.690 | 2.993 ± 0.489 | P = 0.005 | 0.225 ± 0.117 | 1.261 ± 0.244 | 250 – 500 ms |
| P = <0.001 | 1.007 ± 0.364 | 1.72 ± 0.38 | P = 0.047 | 0.451 ± 0.138 | 0.858 ± 0.129 | 500 – 750 ms |
| P = 0.009 | 1.018 ± 0.285 | 1.654 ± 0.335 | P = 0.640 | 0.614 ± 0.161 | 0.68 ± 0.113 | 750 – 1000 ms |
| P = 0.046 | 0.691 ± 0.17 | 1.258 ± 0.307 | P = 0.079 | 0.342 ± 0.12 | 0.709 ± 0.155 | 1000 – 1250 ms |

## Slide 3
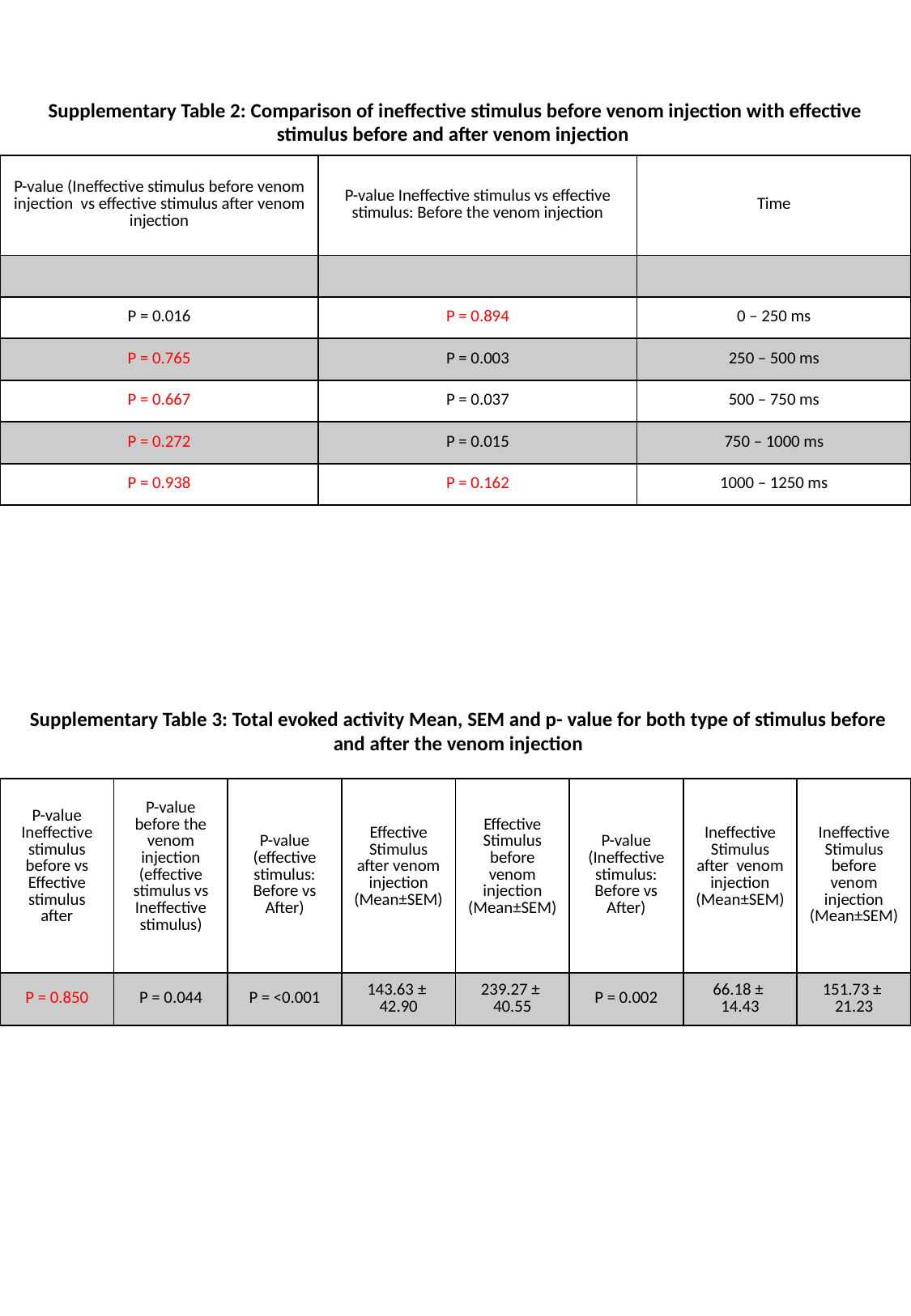

Supplementary Table 2: Comparison of ineffective stimulus before venom injection with effective stimulus before and after venom injection
| P-value (Ineffective stimulus before venom injection vs effective stimulus after venom injection | P-value Ineffective stimulus vs effective stimulus: Before the venom injection | Time |
| --- | --- | --- |
| | | |
| P = 0.016 | P = 0.894 | 0 – 250 ms |
| P = 0.765 | P = 0.003 | 250 – 500 ms |
| P = 0.667 | P = 0.037 | 500 – 750 ms |
| P = 0.272 | P = 0.015 | 750 – 1000 ms |
| P = 0.938 | P = 0.162 | 1000 – 1250 ms |
Supplementary Table 3: Total evoked activity Mean, SEM and p- value for both type of stimulus before and after the venom injection
| P-value Ineffective stimulus before vs Effective stimulus after | P-value before the venom injection (effective stimulus vs Ineffective stimulus) | P-value (effective stimulus: Before vs After) | Effective Stimulus after venom injection (Mean±SEM) | Effective Stimulus before venom injection (Mean±SEM) | P-value (Ineffective stimulus: Before vs After) | Ineffective Stimulus after venom injection (Mean±SEM) | Ineffective Stimulus before venom injection (Mean±SEM) |
| --- | --- | --- | --- | --- | --- | --- | --- |
| P = 0.850 | P = 0.044 | P = <0.001 | 143.63 ± 42.90 | 239.27 ± 40.55 | P = 0.002 | 66.18 ± 14.43 | 151.73 ± 21.23 |

## Slide 4
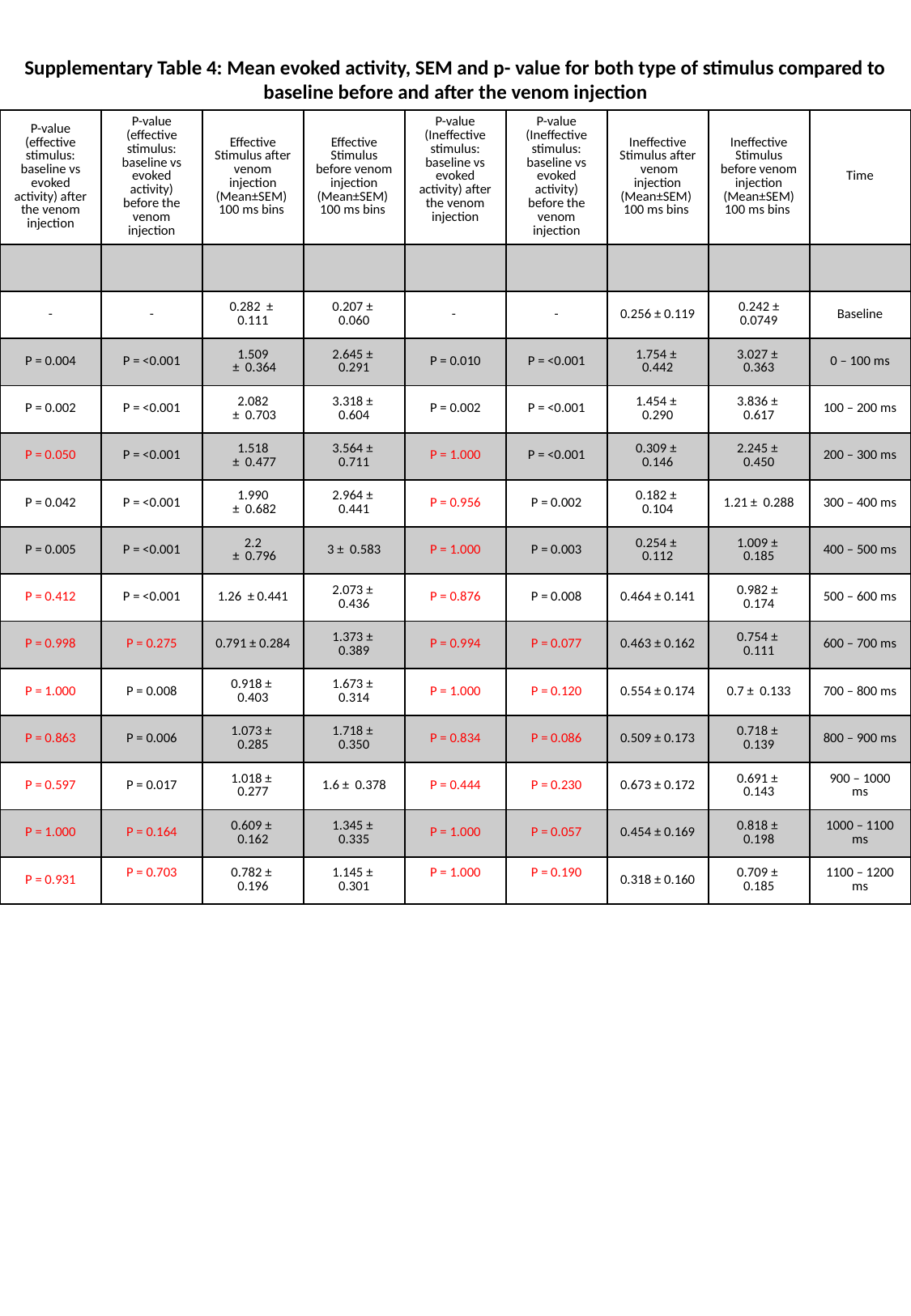

Supplementary Table 4: Mean evoked activity, SEM and p- value for both type of stimulus compared to baseline before and after the venom injection
| P-value (effective stimulus: baseline vs evoked activity) after the venom injection | P-value (effective stimulus: baseline vs evoked activity) before the venom injection | Effective Stimulus after venom injection (Mean±SEM) 100 ms bins | Effective Stimulus before venom injection (Mean±SEM) 100 ms bins | P-value (Ineffective stimulus: baseline vs evoked activity) after the venom injection | P-value (Ineffective stimulus: baseline vs evoked activity) before the venom injection | Ineffective Stimulus after venom injection (Mean±SEM) 100 ms bins | Ineffective Stimulus before venom injection (Mean±SEM) 100 ms bins | Time |
| --- | --- | --- | --- | --- | --- | --- | --- | --- |
| | | | | | | | | |
| - | - | 0.282 ± 0.111 | 0.207 ± 0.060 | - | - | 0.256 ± 0.119 | 0.242 ± 0.0749 | Baseline |
| P = 0.004 | P = <0.001 | 1.509 ± 0.364 | 2.645 ± 0.291 | P = 0.010 | P = <0.001 | 1.754 ± 0.442 | 3.027 ± 0.363 | 0 – 100 ms |
| P = 0.002 | P = <0.001 | 2.082 ± 0.703 | 3.318 ± 0.604 | P = 0.002 | P = <0.001 | 1.454 ± 0.290 | 3.836 ± 0.617 | 100 – 200 ms |
| P = 0.050 | P = <0.001 | 1.518 ± 0.477 | 3.564 ± 0.711 | P = 1.000 | P = <0.001 | 0.309 ± 0.146 | 2.245 ± 0.450 | 200 – 300 ms |
| P = 0.042 | P = <0.001 | 1.990 ± 0.682 | 2.964 ± 0.441 | P = 0.956 | P = 0.002 | 0.182 ± 0.104 | 1.21 ± 0.288 | 300 – 400 ms |
| P = 0.005 | P = <0.001 | 2.2 ± 0.796 | 3 ± 0.583 | P = 1.000 | P = 0.003 | 0.254 ± 0.112 | 1.009 ± 0.185 | 400 – 500 ms |
| P = 0.412 | P = <0.001 | 1.26 ± 0.441 | 2.073 ± 0.436 | P = 0.876 | P = 0.008 | 0.464 ± 0.141 | 0.982 ± 0.174 | 500 – 600 ms |
| P = 0.998 | P = 0.275 | 0.791 ± 0.284 | 1.373 ± 0.389 | P = 0.994 | P = 0.077 | 0.463 ± 0.162 | 0.754 ± 0.111 | 600 – 700 ms |
| P = 1.000 | P = 0.008 | 0.918 ± 0.403 | 1.673 ± 0.314 | P = 1.000 | P = 0.120 | 0.554 ± 0.174 | 0.7 ± 0.133 | 700 – 800 ms |
| P = 0.863 | P = 0.006 | 1.073 ± 0.285 | 1.718 ± 0.350 | P = 0.834 | P = 0.086 | 0.509 ± 0.173 | 0.718 ± 0.139 | 800 – 900 ms |
| P = 0.597 | P = 0.017 | 1.018 ± 0.277 | 1.6 ± 0.378 | P = 0.444 | P = 0.230 | 0.673 ± 0.172 | 0.691 ± 0.143 | 900 – 1000 ms |
| P = 1.000 | P = 0.164 | 0.609 ± 0.162 | 1.345 ± 0.335 | P = 1.000 | P = 0.057 | 0.454 ± 0.169 | 0.818 ± 0.198 | 1000 – 1100 ms |
| P = 0.931 | P = 0.703 | 0.782 ± 0.196 | 1.145 ± 0.301 | P = 1.000 | P = 0.190 | 0.318 ± 0.160 | 0.709 ± 0.185 | 1100 – 1200 ms |
